# Genetic variants in *HLA-DQA1/DQB1* genes modulate the risk of gestational diabetes mellitus in a southern Chinese population

**DOI:** 10.3389/fendo.2025.1511561

**Published:** 2025-07-23

**Authors:** Lijie Nie, Yan Sun, Ruiqi Li, Qiulian Liang, Guocun Deng, Xinyu He, Yifei Zeng, Hui Zheng, Xinhe Xiao, Xiaodong Ding, Jian Huang, Xiangyuan Yu

**Affiliations:** ^1^ The School of Public Health, Guilin Medical University, Guilin, China; ^2^ Institute of Biomedical Research, School of Intelligent Medicine and Biotechnology, Guilin Medical University, Guilin, China; ^3^ Guangxi Key Laboratory of Diabetic Systems Medicine, Guilin Medical University, Guilin, China

**Keywords:** gestational diabetes mellitus, *HLA-DQA1/DQB1*, risk, variant, gene-gene interaction

## Abstract

**Background:**

Gestational diabetes mellitus (GDM) is an endocrine disorder that occurs easily in women during pregnancy. *HLA-DQA1/DQB1* genes play a crucial role in the regulation of the human immune and endocrine systems, potentially influencing the pathogenesis of GDM.

**Objective:**

To explore the associations between single nucleotide polymorphisms (SNPs) in *HLA-DQA1/DQB1* genes and the risk of GDM.

**Methods:**

Seven functional SNPs of *HLA-DQA1/DQB1* genes were selected and genotyped in 523 GDM patients and 638 normal pregnant women. The odds ratio (OR) and its corresponding 95% confidence interval (CI) were utilized to assess the association between candidate SNPs and the risk of GDM. And then, false positive report probability (FPRP), multifactor dimensionality reduction (MDR) and haplotype analysis were employed to validate the statistically significant associations between studied SNPs and GDM risk.

**Results:**

Compared to those with 0–1 risk genotypes, individuals with 2–7 unfavorable genotypes presented an increased risk of GDM (adjusted OR = 1.54, 95% CI=1.04-2.28, *P*=0.033). A dose- accumulation effect was detected between the number of unfavorable genotypes and GDM risk (*P*_trend_=0.024). Furthermore, stratified analysis revealed that the increased GDM risk was more likely to occur in individuals with higher blood pressure and TG, and lower HDL-c levels (*P*<0.05). Multifactor dimensionality reduction (MDR) analysis revealed that rs9274666 was the best single locus model, whereas the 7-loci model was the best multifactor interaction model for predicting GDM risk (χ²=134.28, *P*<0.0001). Finally, haplotype analysis revealed that the ACGAGTA and ACGGATA haplotypes were significantly associated with the increased GDM risk. *HLA-DQA1/DQB1* SNPs can significantly alter individuals’ genetic susceptibility to GDM.

**Conclusions:**

The genetic variations in the *HLA-DQA1* and *HLA-DQB1* genes may collectively contribute to the susceptibility to gestational diabetes mellitus. These findings suggest that these genetic markers could be useful for early prediction of GDM, and further validation in larger cohorts is warranted.

## 1 Introduction

Gestational diabetes mellitus (GDM) is defined as any degree of glucose intolerance first recognized during pregnancy, typically diagnosed through an oral glucose tolerance test during the second trimester. Globally, the incidence of GDM ranges from 2% to 25% (1, 2). As a major complication during pregnancy, GDM not only increases reproductive risk for expectant mothers but also has potential genetic implications for offspring. These genetic effects may increase the risk of chronic conditions such as obesity and diabetes, and lead to abnormal fetal growth patterns, including both excessive and restricted growth (3, 4). Despite its significant impact, the pathogenesis of GDM remains complex and has not yet been fully elucidated.

Clinically, GDM primarily arises from the persistent insulin resistance in pregnant women. Additional risk factors include advanced maternal age, a family history of diabetes, a previous history of GDM, immune factors, overweight or obesity, dietary habits, and physical inactivity (5–7). Given these risk factors, recent research has shifted toward exploring novel contributors to the prevalence of GDM, particularly genetic and epigenetic factors, including gene-environment interactions (7–9). Understanding genetic susceptibility is crucial, as it can lead to early identification and targeted interventions for at-risk populations.

Numerous studies have aimed to clarify the specific impact and risk levels associated with genetic variations in the development of GDM. Single nucleotide polymorphisms (SNPs), which are variations at a single position in a DNA sequence among individuals, provide critical information about genetic predispositions to complex diseases (10). These genetic variations may directly influence GDM risk or interact with clinical traits such as age, pre-pregnancy body mass index (pre-BMI), insulin secretion, pancreatic β-cell function, and blood glucose levels (4, 11–13). To date, genome-wide association studies and candidate gene studies have identified numerous SNPs associated with GDM (10, 14), offering new insights into its pathogenesis.

The human leucocyte antigen (HLA) gene complex plays a pivotal role in regulating the immune system and is characterized by high genetic diversity (15, 16). Various HLA alleles have been linked to susceptibility to different types of diabetes (17, 18). For example, type 1 diabetes mellitus (T1DM) is an autoimmune disorder with a substantial genetic component, where HLA class II gene variants such as DQA1*0301, DQB1*0302, DQA1*0501, DQB1*0201, DQB1*060, DQB1*0302 (DR4), DRB1*03 and DRB1*04 account for 30-50% of its heritability (10, 19, 20). Similarly, specific *HLA-DQA1* and *HLA-DQB1* genes have been associated with susceptibility to type 2 diabetes mellitus (T2DM) (21, 22). Although studies have explored the relationship between HLA-related genes and genetic susceptibility to GDM, identifying significant polymorphisms such as HLA-DQB10602 are being negatively associated with GDM risk (3, 23), the role of SNPs in the *HLA-DQA1* and *HLA-DQB1* genes in GDM pathogenesis remains unclear.

In this case-control study involving 523 GDM women and 638 normoglycemic pregnant women, we genotyped seven SNPs in the *HLA-DQA1* and *HLA-DQB1* genes, which were previously identified via the illumina Asian Screening Array (ASA) BeadChip. Our objective was to investigate the associations between these genetic variants and susceptibility to GDM. These findings may pave the way for genetic screening tools that identify women at higher risk for GDM, contributing to better prevention and management strategies.

## 2 Materials and methods

### 2.1 Study subjects

**Figure 1:**
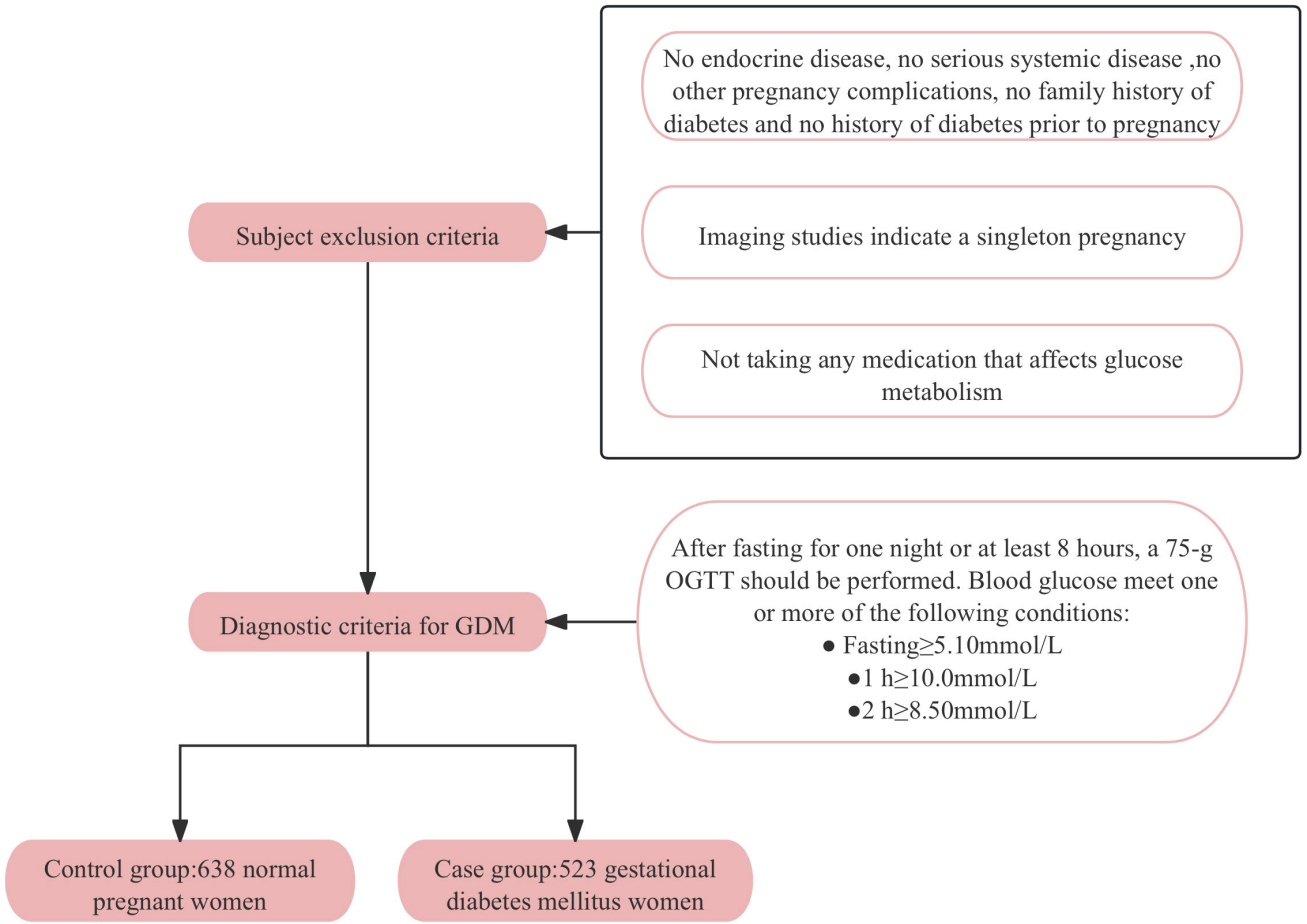
The flow chart of study participant recruitment.

A control group of 638 normoglycemic pregnant women and a case group of 523 GDM women were enrolled at the Affiliated Hospital of Guilin Medical University from September 2014 to April 2016. All participants were genetically unrelated and provided written informed consent. Participants with pre-existing diabetes, chronic diseases, family history of diabetes or pregnancy complications were excluded to ensure a homogenous study population. The diagnostic criteria for GDM followed the standards established by the International Association of Diabetes and Pregnancy Study Groups (IADPSG), which were chosen for their sensitivity and international acceptance. According to these criteria, pregnant women without a previous diagnosis of diabetes underwent a 75-g oral glucose tolerance test (OGTT) after an overnight fast at 24–28 weeks of gestation. A diagnosis of GDM was made if any one or more of the following conditions were met: fasting blood glucose (FBG) ≥ 5.1mmol/L, postprandial 1-hour blood glucose ≥ 10.0mmol/L, or postprandial 2-hour blood glucose ≥ 8.5mmol/L. **Figure 1** provides a detailed flowchart of participant recruitment and selection criteria. All participants have signed informed consent forms and the research protocol has been approved by the Ethics Committee of Guilin Medical University (Number: GLMC20131205).

### 2.2 Asian Screening Array

The DNA extraction kit (Tiangen Biotech) was used to extract DNA from the samples, and then the gene typing module of Genomestudio v2.1 (Illumina) was subsequently used to obtain high-quality data suitable for genome-wide association studies. In the discovery phase, the dataset needs to meet the following criteria: (1) SNP call rate > 95%, in Hardy-Weinberg equilibrium (HWE); (2) sample call rate > 95%; and (3) minimum allele frequency (MAF) < 1%.

### 2.3 Candidate variant selection

On the basis of the ASA Bead Chip, at the statistical level of 5*10^-4^, SNPs were screened. In reference to the NCBI (http://www.ncbi.nlm.nih.gov/projects/SNP) and SNP info web server (http://snpinfo.niehs.nih.gov/), potential SNPs are located in the functional regions of the *HLA-DQA1* and *HLA-DQB1* genes, such as gene regulatory regions, microRNA binding site (MBS), transcription factor binding site (TFBS), splicing site (SS), etc. with minor allele frequency (MAF) greater than 5% in the Chinese Han Beijing population; Finally, seven SNPs, namely, rs1391371, rs9272425, rs9272426, rs9272460, rs9273368, rs9273505 and rs9274666 were selected.

### 2.4 Clinical and biochemical data

Comprehensive data on age, pre-pregnancy body mass index (pre-BMI), systolic blood pressure (SBP), diastolic blood pressure (DBP), and biochemical parameters were systematically collected from all participants. For biochemical analyses of triglyceride (TG), total cholesterol (TC), and high-density lipoprotein cholesterol (HDL-c) levels, blood samples were collected in no anticoagulant tubes after the participants had fasted for at least 12 hours. Blood samples for fasting blood glucose (FBG), 1-hour OGTT, and 2-hour OGTT were collected in tubes containing sodium fluoride-potassium oxalate as an anticoagulant to inhibit glycolysis. Hemoglobin A1c (HbA1c) testing requires blood samples collected in tubes containing EDTA as an anticoagulant. A minimum of 3 milliliters of blood were collected for each test to ensure sufficient volume for accurate analysis. All biochemical assays were performed in the hospital’s clinical laboratory via standardized procedures and calibrated equipment to ensure the reliability and validity.

### 2.5 Genomic DNA extraction and genotyping

Genomic DNA was extracted from EDTA-anticoagulated whole blood samples via a commercial DNA extraction kit (Aidlab Biotechnologies Co., Ltd., China) according to the manufacturer’s protocol. The concentration of the extracted DNA was assessed by measuring the absorbance at 260 nm and 280 nm via a UV spectrophotometer, ensuring that the A260/A280 ratio was between 1.80 and 2.00 for optimal quality.

The specific amplification primers used for the selected SNPs are listed in **Supplementary Table S1**. The total volume of the PCR system was 5 μL, including 1 μL of template DNA (20~100 ng/μL), 1.850 μL of ddH_2_O, 0.625 μL of 1.25×PCR buffer (15 mmol/L MgCl_2_), 0.325 μL of 25 mmol/L MgCl_2_, 0.1 μL of 25 mmol/L dNTP mixture, 1 μL of 0.5 μmol/L primer mixture, and 0.1 μL of 5 U/μL HotStar Taq polymerase. The PCR amplification program was as follows: first, 15 minutes of predenaturation at 94°C was performed, followed by 45 cycles. Each cycle included denaturation at 94°C for 20 seconds, annealing at 56°C for 30 seconds, and extension at 72°C for 1 minute. Finally, a final incubation was carried out at 72°C for 3 minutes.

The amplified PCR products were analyzed via the Sequenom MassARRAY platform, which employs matrix-assisted laser desorption/ionization time-of-flight mass spectrometry (MALDI-TOF MS) for SNP genotyping. The genotype data were processed and analyzed via TYPER 4.0 software. Representative genotyping scatter plots are presented in **Supplementary Figure S1**, demonstrating the reliability and accuracy of the genotyping.

### 2.6 Statistical analysis

The changes in the clinical and biochemical test data are represented as the mean (SD), whereas the differences in the demographic variables were evaluated using chi-square tests. The Hardy-Weinberg equilibrium of the genotype distribution was tested using chi-square (χ^2^) goodness-of-fit test. Binary logistic regression analysis was used to assess the risk of GDM by calculating odds ratios (ORs) and their corresponding 95% confidence intervals (CIs). All statistical analyses were performed using the IBM SPSS Statistics 28 for Windows (IBM Corp, Armonk, NY, USA) with two-tailed tests, and *P*<0.05 was considered statistically significant.

Multifactor Dimensionality Reduction (MDR) software (version 3.0.2) was used to identifying interaction effects across the studied SNPs and construct the best multifactor model through 1000 permutations under 100-fold cross-validation consistency (CVC) without associations.

Linkage disequilibrium (LD) analysis of each SNP locus and construction of haplotypes were conducted using SHEsis software (http://analysis.bio-x.cn/) (24, 25). SNPs were considered to exhibit strong linkage when r^2^ > 0.6 and D′ > 0.7, moderate linkage when 0.3≤ r^2^ ≤ 0.6 and 0.4 ≤ D′ ≤ 0.7, and weak linkage when r^2^ < 0.3 and D′ < 0.4 (26). Understanding the LD between SNPs allows for the identification of haplotypes that may be associated with GDM risk.

The false positive report probability (FPRP) was estimated to assess the robustness of findings with a statistically significant association with a prior probability set at 0.1 and a relatively strict cutoff value of 0.2 (27).

## 3 Results

### 3.1 Characteristics of the subjects

This study included a total of 1161 pregnant women. A comparison between the control group and the GDM group revealed that pregnant women with GDM had significantly higher values for age, pre-BMI, SBP, DBP, TG, HbA1c, FPG, and 1-hour and 2-hour OGTT than those of the normoglycemic pregnant women (*P*<0.05), as shown in **Table 1**.

**Table 1:** Characteristics of GDM cases and controls.

Variables	Controls (n=638), mean (SD)	Cases (n=523), mean (SD)	*t*	*P*
Age (years)	28.90 ± 4.15	31.43 ± 4.78	9.50	**<0.001** ^***^
pre-BMI (kg/m^2^)	21.47 ± 2.30	23.14 ± 3.65	8.41	**<0.001** ^***^
SBP (mmHg)	108.76 ± 9.36	111.59 ± 10.66	4.74	**<0.001** ^***^
DBP (mmHg)	68.57 ± 8.29	70.51 ± 8.28	3.98	**<0.001** ^***^
TG (mmol/L)	2.43 ± 1.00	2.65 ± 1.20	3.48	**0.001** ^***^
TC (mmol/L)	5.30 ± 1.09	5.35 ± 1.15	0.75	0.452
HDL-c (mmol/L)	1.65 ± 0.40	1.66 ± 0.42	0.50	0.616
HbA1c (%)	5.00 ± 0.49	5.44 ± 0.69	12.38	**<0.001** ^***^
FBG (mmol/L)	4.40 ± 0.33	5.26 ± 1.35	14.29	**<0.001** ^***^
OGTT 1h glucose (mmol/L)	6.96 ± 1.41	9.80 ± 2.25	25.14	**<0.001** ^***^
OGTT 2h glucose (mmol/L)	6.08 ± 1.09	8.34 ± 2.16	21.73	**<0.001** ^***^

Significance: ^*^ *P* < 0.05; ^**^ *P* < 0.01; ^***^ *P* < 0.001.

Bold values: statistically significant.

In the term of the association between genetic variant genotype and GDM risk, the genotype frequencies of the four loci of *HLA-DQA1* and the three loci of *HLA-DQB1* were all in accordance with the Hardy-Weinberg equilibrium as shown in **Table 2**.

**Table 2:** Effects of risk genotypes of *HLA-DQA1/DQB1* variation GDM.

Genotype	Controls, n (%)	Cases, n (%)	*P* ^a^	Crude OR (95%CI)^b^	*P* ^b^	Adjusted OR (95% CI)^c^	*P* ^c^
HLA-DQA1							
rs1391371							
AA	132 (22.5)	96 (19.6)	0.323	1		1	
AT	33 (5.6)	22 (4.5)		0.92 (0.50-1.67)	0.776	1.07 (0.57-2.01)	0.844
TT	422 (71.9)	371 (75.9)		1.21 (0.90-1.62)	0.212	1.24 (0.90-1.70)	0.190
AT/TT	455 (77.5)	393 (80.4)	0.254	1.19 (0.88-1.60)	0.254	1.23 (0.89-1.68)	0.207
AA/AT	165 (28.1)	118 (24.1)		1			
TT	422 (71.9)	371 (75.9)	0.140	0.81 (0.62-1.07)	0.140	1.22 (0.91-1.64)	0.180
rs9272425							
TT	362 (60.1)	308 (61.6)	0.677	1		1	
TC	114 (18.9)	98 (19.6)		1.01 (0.74-1.38)	0.948	0.96 (0.69-1.34)	0.830
CC	126 (20.9)	94 (18.8)		0.88 (0.65-1.19)	0.402	0.83 (0.60-1.16)	0.272
TC/CC	240 (39.9)	192 (38.4)	0.619	0.94 (0.74-1.20)	0.619	0.90 (0.69-1.16)	0.402
TT/TC	476 (79.1)	406 (81.2)		1			
CC	126 (20.9)	94 (18.8)	0.378	0.88 (0.65-1.12)	0.379	0.84 (0.61-1.15)	0.281
rs9272426							
AA	243 (39.3)	227 (44.0)	0.268	1		1	
AG	208 (33.7)	164 (31.8)		0.84 (0.64-1.11)	0.224	0.81 (0.61-1.08)	0.153
GG	167 (27.0)	125 (24.2)		0.80 (0.60-1.08)	0.140	0.78 (0.57-1.07)	0.122
AG/GG	375 (60.7)	289 (56.0)	0.112	0.83 (0.65-1.05)	0.112	0.80 (0.62-1.03)	0.077
AA/AG	451 (73.0)	391 (75.8)		1			
GG	167 (27.0)	125 (24.2)	0.283	0.86 (0.66-1.13)	0.283	0.86 (0.65-1.14)	0.292
rs9272460							
AA	380 (63.8)	323 (68.3)	0.266	1		1	
AG	138 (23.2)	92 (19.5)		0.78 (0.58-1.06)	0.116	0.80 (0.58-1.11)	0.178
GG	78 (13.1)	58 (12.3)		0.88 (0.60-1.27)	0.480	0.83 (0.56-1.23)	0.356
AG/GG	216 (36.2)	150 (31.7)	0.121	0.82 (0.63-1.06)	0.121	0.81 (0.62-1.07)	0.134
AA/AG	518 (86.9)	415 (87.7)		1			
GG	78 (13.1)	58 (12.3)	0.688	0.93 (0.65-1.34)	0.688	0.88 (0.60-1.29)	0.503
HLA-DQB1							
rs9273368							
AA	55 (8.9)	47 (9.4)	0.329	1		1	
AG	243 (39.4)	176 (35.1)		0.85 (0.55-1.31)	0.456	0.93 (0.58-1.49)	0.765
GG	319 (51.7)	279 (55.6)		1.02 (0.67-1.56)	0.914	1.12 (0.71-1.77)	0.619
AG/GG	562 (91.1)	455 (90.6)	0.795	0.95 (0.63-1.43)	0.795	1.04 (0.67-1.61)	0.865
AA/AG	298 (48.3)	223 (44.4)		1			
GG	319 (51.7)	279 (55.6)	0.196	1.17 (0.92-1.48)	0.196	1.19 (0.92-1.53)	0.180
rs9273505							
CC	348 (58.8)	287 (59.1)	0.060	1		1	
CT	64 (10.8)	73 (15.0)		1.38 (0.96-2.00)	0.086	1.28 (0.87-1.91)	0.215
TT	180 (30.4)	126 (25.9)		0.85 (0.64-1.12)	0.245	0.79 (0.59-1.06)	0.123
CT/TT	244 (41.2)	199 (40.9)	0.929	0.99 (0.78-1.26)	0.929	0.92 (0.71-1.20)	0.537
CC/CT	412 (69.6)	360 (74.1)		1			
TT	180 (30.4)	126 (25.9)	0.105	0.80 (0.61-1.05)	0.105	0.76 (0.57-1.01)	0.058
rs9274666							
AA	209 (36.9)	169 (34.9)	0.168	1		1	
AG	133 (23.5)	97 (20.0)		0.90 (0.65-1.26)	0.541	0.97 (0.68-1.38)	0.857
GG	224 (39.6)	218 (45.0)		1.20 (0.91-1.59)	0.187	1.25 (0.93-1.68)	0.134
AG/GG	357 (63.1)	315 (65.1)	0.499	1.09 (0.85-1.40)	0.499	1.15 (0.88-1.50)	0.319
AA/AG	342 (60.4)	266 (55.0)		1			
GG	224 (39.6)	218 (45.0)	0.074	1.25 (0.98-1.60)	0.074	1.27 (0.98-1.65)	0.077
Number of risk genotypes							
0-1	88 (20.6)	54 (14.8)	0.243	1		1	
2	13 (3.0)	10 (2.7)		1.25 (0.51-3.06)	0.619	1.45 (0.56-3.71)	0.442
3	33 (7.7)	29 (8.0)		1.43 (0.78-2.62)	0.243	1.62 (0.85-3.07)	0.142
4	81 (18.9)	64 (17.6)		1.29 (0.80-2.06)	0.293	1.25 (0.76-2.07)	0.381
5	76 (17.8)	71 (19.5)		1.52 (0.95-2.43)	0.079	1.61 (0.98-2.66)	0.061
6	109 (25.5)	98 (26.9)		1.47 (0.95-2.26)	0.085	1.49 (0.94-2.37)	0.091
7	28 (6.5)	38 (10.4)		2.21 (1.22-4.01)	**0.009** ^**^	2.34 (1.24-4.41)	**0.008** ^**^
Trend					**0.020** ^*^		**0.024** ^*^
Dichotomized groups							
0-1	88 (20.6)	54 (14.8)	**0.036** ^*^	1			
2-7	340 (79.4)	310 (85.2)		1.49 (1.02-2.16)	**0.037** ^*^	1.54 (1.04-2.28)	**0.033** ^*^

a Two-sided χ^2^ test for genotypes distributions between cases and controls.

b Unconditional logistic regression analysis.

c Adjusted for age, pre-BMI in logistic regression models.

Significance: ^*^ *P* < 0.05; ^**^ *P* < 0.01; ^***^ *P* < 0.001.

Bold values: statistically significant.

Given the lack of significant associations with individual genotypes, we conducted a combined- genotype effect analysis based on the basis of the number of risk genotypes. All putative risk genotypes, those with an odds ratio (OR) greater than 1.0 (and for OR < 1.0, the reference group was reversed), from the studied SNPs were categorized into new variables according to the number of risk genotypes (28). The risk genotypes used for the calculation were *HLA-DQA1* gene rs1391371 AT/TT, rs9272425 TT, rs9272426 AA and rs99272460 AA, and *HLA-DQB1* gene rs9273368 GG, rs9273505 CT and rs9274666 AG/GG.

As shown in **Table 2**, we observed that the likelihood of developing GDM increased with the number of risk genotypes. Compared with individuals with 0–1 risk genotypes, those carrying all 7 unfavorable genotypes had a significantly increased risk of GDM (adjusted OR = 2.34, 95% CI = 1.24-4.41, *P*=0.008). Additionally, individuals with 2–7 unfavorable genotypes presented increased GDM risk (adjusted OR = 1.54, 95% CI = 1.04-2.28, *P*= 0.033) compared with those with 0–1 risk genotypes. Notably, this cumulative effect on GDM risk appeared to be dose-dependent with respect to the number of risk genotypes (*P*_trend_ = 0.024).

### 3.2 Stratified analysis of risk genotypes

We conducted a stratified analysis to assess the association between the number of risk genotypes (2–7 unfavorable genotypes) and GDM risk within subgroups defined by the mean values of clinical variables. As shown in **Table 3**, the increased GDM risk associated with carrying 2–7 unfavorable genotypes was more pronounced among participants with TG > 2.53 (OR=1.94, 95% CI=1.10-3.42, *P*=0.022), age ≤ 30.04 (adjusted OR=1.85, 95% CI=1.05-3.27, *P*=0.033), SBP >110.03 (adjusted OR=1.83, 95% CI=1.00-3.33, *P*= 0.050), DBP > 69.44 (adjusted OR=2.11, 95% CI=1.12-3.96, *P*=0.021) and HDL-c ≤ 1.65 (adjusted OR=1.86, 95%CI=1.07-3.22, *P*=0.028).

**Table 3:** Stratification analysis for associations between risk genotypes of *HLA-DQA1*/*DQB1* and GDM risk.

Variables	0–1 Risk Genotype (Cases/Controls)	2–7 Risk Genotypes (Cases/Controls)	Crude OR (95%CI)^a^	*P* ^a^	Adjusted OR (95%CI)^b^	*P* ^b^	*P* ^c^
Age (years)							
≤30.04	21/61	140/232	**1.75 (1.02-3.00)**	**0.041** ^*^	**1.85 (1.05-3.27)**	**0.033** ^*^	0.910
>30.04	33/27	170/108	1.29 (0.73-2.26)	0.378	1.29 (0.73-2.29)	0.382	
pre-BMI (kg/m^2^)							
≤22.22	22/62	128/231	1.56 (0.92-2.66)	0.101	1.68 (0.96-2.94)	0.068	0.393
>22.22	32/26	182/109	1.36 (0.77-2.40)	0.294	1.39 (0.78-2.48)	0.267	
SBP (mmHg)							
≤110.03	29/51	147/186	1.39 (0.84-2.30)	0.201	1.35 (0.80-2.29)	0.262	0.270
>110.03	25/37	163/154	1.57 (0.90-2.72)	0.112	**1.83 (1.00-3.33)**	**0.050** ^*^	
DBP (mmHg)							
≤69.44	33/51	147/177	1.57 (0.90-2.72)	0.112	1.28 (0.77-2.12)	0.349	0.044
>69.44	21/37	163/163	1.76 (0.99-3.14)	0.055	**2.11 (1.12-3.96)**	**0.021** ^*^	
TG (mmol/L)							
≤2.53	31/50	154/207	1.20 (0.73-1.97)	0.470	1.35 (0.80-2.31)	0.264	0.744
>2.53	23/38	156/133	**1.94 (1.10-3.42)**	**0.022** ^*^	1.78 (0.99-3.22)	0.055	
TC (mmol/L)							
≤5.33	30/52	148/172	1.49 (0.90-2.46)	0.117	1.59 (0.93-2.71)	0.088	0.066
>5.33	24/36	162/168	1.45 (0.83-2.53)	0.196	1.43 (0.79-2.58)	0.243	
HDL-c (mmol/L)							
≤1.65	27/53	150/177	1.66 (1.00-2.78)	0.051	**1.86 (1.07-3.22)**	**0.028** ^*^	0.193
>1.65	27/35	160/163	1.27 (0.74-2.20)	0.388	1.23 (0.69-2.18)	0.484	
HbA1c (%)							
≤5.20	23/68	114/258	1.31 (0.78-2.20)	0.315	1.33 (0.78-2.26)	0.301	0.133
>5.20	31/20	196/82	1.54 (0.83-2.86)	0.170	1.65 (0.85-3.17)	0.137	

a Unconditional logistic regression analysis.

b Adjusted for age, pre-BMI in logistic regression models.

c Homogeneity test using the χ^2^.

Significance: ^*^ *P* < 0.05; ^**^ *P* < 0.01; ^***^ *P* < 0.001.

Bold values: statistically significant.

### 3.3 FPRP analysis

The FPRP test was used to assess whether there was a significant correlation between the accumulation of multiple unfavorable genes in SNPs and the risk of GDM in this study. See **Table 4**, the FPRP value for the association between seven unfavorable genotypes and GDM risk is 0.191 (less than the threshold value of 0.20), indicating that association is authentic.

**Table 4:** FPRP analysis for the positive associations of *HLA-DQA1/DQB1* variants and GDM risk.

Combined risk genotypes	Adjusted OR (95% CI)	*P*	Statistical Power	Prior probability					
				0.25	0.1	0.01	0.001	0.0001	0.00001
7 vs.0-1	2.34 (1.24-4.41)	0.008	0.081	0.073	**0.191**	0.722	0.963	0.996	1.000
2–7 vs.0-1	1.54 (1.04-2.28)	0.033	0.457	0.178	0.394	0.877	0.986	0.999	1.000
Subgroup									
Age ≤ 30.04	1.85 (1.05-3.27)	0.033	0.600	0.142	0.331	0.845	0.982	0.998	1.000
SBP>110.03	1.83 (1.00-3.33)	0.050	0.621	0.194	0.420	0.888	0.988	0.999	1.000
DBP>69.44	2.11 (1.12-3.96)	0.021	0.440	0.1225	0.300	0.825	0.979	0.998	1.000
HDL-c ≤ 1.65	1.86 (1.07-3.22)	0.028	0.567	0.100	0.250	0.786	0.974	0.997	1.000

Bold values indicate that the difference is statistically significant at the test level of FPRP=0.2.

### 3.4 Gene-gene interaction effects on GDM risk according

In the MDR analysis, we identified the best single-locus model as rs9274666 in **Table 5**, which is significantly associated with GDM risk (χ^2^ = 5.27, *P*=0.0217) and achieved the highest CVC of 100/100 and a balanced testing accuracy of 0.5195. More interestingly, the model with all seven studied SNPs with a whole balanced accuracy of 0.6636 and a maximum CVC of 100/100 was the best model for evaluate the GDM risk, with a χ^2^ test of 134.28 and the corresponding *P*<0.0001.

**Table 5:** MDR analysis for the GDM risk prediction.

Best model	Training balanced accuracy	Testing balanced accuracy	Whole balanced accuracy	CVC	χ^2^	*P*
1	0.5329	0.5195	0.5329	100/100	5.27	**0.0217** ^*^
1,2	0.5487	0.5256	0.5487	92/100	11.14	**<0.001** ^***^
1,2,3	0.5741	0.4677	0.5736	79/100	25.10	**<0.001** ^***^
1,2,3,4	0.6056	0.4602	0.6050	100/100	50.79	<0.001^***^
1,2,3,4,5,	0.6321	0.4552	0.6316	95/100	83.22	<0.001^***^
1,2,3,4,5,6	0.6572	0.4906	0.6568	100/100	121.16	<0.001^***^
1,2,3,4,5,6,7	0.6640	0.4963	0.6636	100/100	134.28	<0.001^***^

Labels: 1:rs9274666, 2: rs9272426, 3:rs9272460, 4:rs9273505, 5:rs9272425, 6:rs9273368, 7:rs1391371.

Significance: ^*^ *P* < 0.05; ^**^ *P* < 0.01; ^***^ *P* < 0.001.

Bold values: statistically significant.

### 3.5 LD analysis

**Figure 2:**
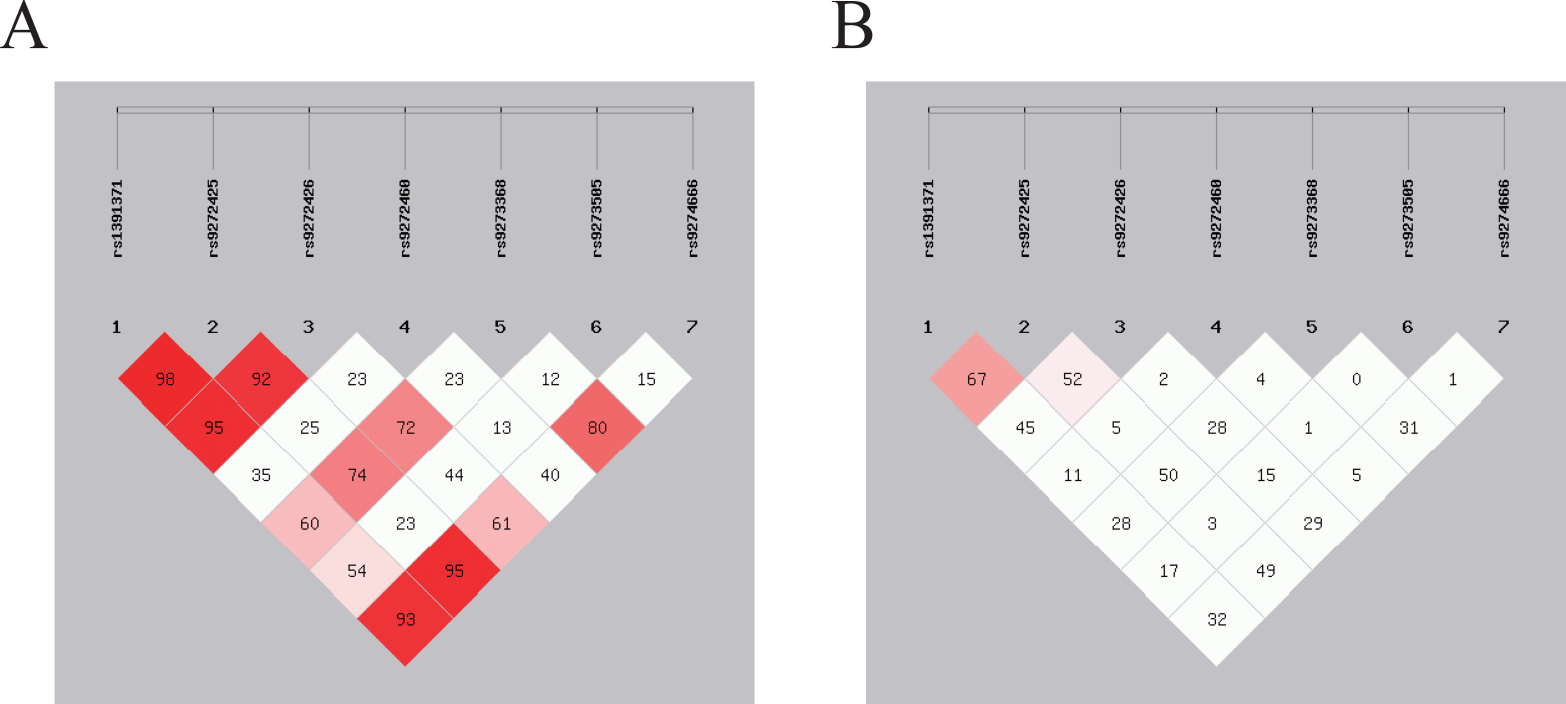
SNPs loci linkage disequilibrium analysis. The strength of LD between SNP pairs, with D’ and r^2^ values representing the degree of genetic correlation. **(A)** The D’ value in the box reflects the overall linkage situation of multi-locus chromosome blocks; **(B)** The r^2^ value in the box reflects the estimated situation of linkage disequilibrium between two loci.

For seven SNPs in *HLA-DQA1/DQB1*, we observed strong LD between rs9272425 and rs1391371 (D′=0.982, r^2^ = 0.671) in **Figure 2**. Moderate LD was found between rs9272426 and both rs1391371 (D′=0.954, r^2^ = 0.458) and rs9272425 (D′=0.929, r^2^ = 0.521), and between rs9274666 and both rs1391371 (D′=0.932, r^2^ = 0.322) and rs9272425 (D′=0.954, r^2^ = 0.491). LD among the other SNPs was relatively weak.

### 3.6 Haplotype analysis

As shown in **Table 6**, when constructing haplotypes for 7 SNP loci within *HLA-DQA1* and *HLA -DQB1* for the GDM group and the control group, we found that the haplotype TTAAGCG was the most common, with frequencies of 35.5% in the GDM group and 33.2% in the control group. An increased risk of GDM may be associated with the haplotypes ACGAGTA (OR=3.31, 95% CI=1.70-6.44, *P*<0.001) and ACGGATA (OR=9.42,95% CI=3.49-25.44, *P*<0.001); whereas the haplotypes ACGAATA (OR=0.13, 95% CI=0.07-0.28, *P*<0.001) and ACGGGTA (OR=0.06, 95% CI=0.02-0.22, *P*<0.001) present a decreased risk effect of GDM.

**Table 6:** Distribution of haplotypes in GDM group and control group.

Haplotypes	GDM, n (%)	Control, n (%)	χ^2^	*P*	OR (95%CI)
**A C G A A T A**	8.51 (1.2)	66.90 (7.8)	39.87	**<0.001** ^***^	**0.13 (0.07~0.28)**
**A C G A G T A**	33.49 (4.6)	12.14 (1.4)	13.80	**<0.001** ^***^	**3.31 (1.70~6.44)**
A C G G A C A	26.00 (3.6)	33.19 (3.9)	0.16	0.689	0.90 (0.53~1.52)
**A C G G A T A**	34.49 (4.7)	4.45 (0.5)	28.63	**<0.001** ^***^	**9.42(3.49~25.44)**
**A C G G G T A**	2.51 (0.3)	44.50 (5.2)	33.18	**<0.001** ^***^	**0.06 (0.02~0.22)**
T C G A A C A	58.97 (8.1)	58.65 (6.9)	0.70	0.402	1.18 (0.81~1.72)
T T A A G C A	60.77 (8.3)	63.20 (7.4)	0.36	0.548	1.12 (0.77~1.62)
T T A A G C G	258.78 (35.5)	284.53 (33.2)	0.52	0.471	1.09 (0.87~1.36)
T T A A G T G	61.89 (8.5)	54.35 (6.3)	2.37	0.124	1.35 (0.92~1.98)
T T A G G C A	28.85 (4.0)	30.54 (3.6)	0.11	0.737	1.09 (0.65~1.84)
T T A G G C G	19.61 (2.7)	31.90 (3.7)	1.52	0.217	0.70 (0.39~1.24)

Significance: ^*^ *P* < 0.05; ^**^ *P* < 0.01; ^***^ *P* < 0.001.

Bold values: statistically significant.

## 4 Discussion

The result of the present study suggests that genetic factors, such as SNPs (29), are strongly linked to the onset of GDM. In this study, among the seven selected loci of *HLA-DQA1/DQB1* genes (rs1391371, rs9272425, rs9272426, rs9272460, rs9273368, rs9273505, and rs9274666), we also observed that the risk of GDM increased correspondingly with the accumulation of unfavorable genotypes when combined. This cumulative effect was further validated by the results from the multifactor MDR analysis. Identification of these SNPs may enable prediction of GDM risk and allow for early intervention strategies to minimize complications for both the pregnant woman and the newborn (30). These findings are important for the prevention and control of GDM in clinical and public health settings.

Previous studies have shown that HLA class II genes (*HLA-DQA1*, *HLA-DRB1*, *HLA-DPA1*, and *HLA-DQB1*) and their antigens may play roles in immune regulation during GDM development (31). *HLA-DQA1/DQB1*, as key molecules within the HLA class II complex, are associated with the pathogenesis of GDM through interactions with regulatory T cells (Tregs) and effector T cells (32). Consistent with this, we established an association between the combined effects of *HLA-DQA1/DQB1* genetic variant genotypes and an increased risk of GDM. Using logistic regression, MDR, FPRP and haplotype analysis, we confirmed the joint effects between the studied loci. These results suggest that *HLA-DQA1/DQB1* gene variants have significant genetic regulatory effects on the pathogenesis of GDM, potentially altering individuals’ susceptibility to GDM through single-locus effects, multifactor combinations, and gene-environment interactions.

FPRP analysis, plays an important role in molecular epidemiology research, by evaluating the authenticity of research results, helping researchers better understand and control potential false positives in research, and thereby improving the scientific and reliability of research (27). To verify the noteworthiness of the significant associations between the studied variants and GDM risk, a much more stringent FPRP threshold of 0.2 was set, and the 7 risk factors, compared with the 0–1 risk genotype carrying effect, are likely to be authentic. However, the FPRP values detected for other statistically significant associations are much greater than 0.2, indicating that these findings might be possible observations. Thus, the conclusions drawn from here must be considered preliminary and need to be verified in the future.

In exploring gene-environment interactions, we observed that combinations of unfavorable genotypes can significantly increase the risk of GDM in pregnant women under the age of 30.04 years, with SBP exceeding 110.03 mmHg, DBP exceeding 69.44 mmHg, TG greater than 2.53 mmol/L, and HDL-c levels below 1.65 mmol/L. Research has indicated that elevated triglycerides can lead to dyslipidemia, wherein excessive free fatty acids are secreted by abundant adipocytes. This process promotes the overproduction of inflammatory cytokines and oxidative reactions, which can damage pancreatic beta cells or induce insulin resistance, ultimately resulting in abnormal glucose levels. HDL-c, as a crucial lipoprotein, not only influences glucose levels through the aforementioned pathways but also exerts effects through its direct anti-inflammatory properties. Low levels of HDL-c can alter insulin sensitivity and pancreatic insulin secretion (33, 34). Although the complex physiological and pathological mechanisms linking elevated blood pressure to glucose imbalance are not yet clear, studies have shown a close relationship between elevated diastolic blood pressure and insulin resistance and GDM. Researchers thought that the two could interact via the renin-angiotensin-aldosterone and sympathetic nervous systems (35, 36). Lack of gestational weight gain records may affect metabolic assessment. Future studies should incorporate longitudinal anthropometric measurements. These findings provide new insights for further exploration of the relationship between genetic factors and the prediction of GDM and cardiovascular disease. In contrast, while most studies suggest that older age is associated with a greater risk of GDM (37, 38), this research revealed that the combined effect of unfavorable genotypes on GDM risk was more pronounced in individuals under the age of 30.04 years. This could be due to the interaction between genetic susceptibility and early metabolic imbalance, which might lead to the earlier onset of GDM in younger individuals (39). Beyond genetic risk stratification, innovative cardiac imaging modalities such as speckle tracking echocardiography (STE) could provide critical insights for identifying among pregnant women those with increased risk of developing GDM or cardiovascular complications (40, 41). It is likely that several genetic variants may serve as predictors of impaired myocardial deformation indices or subclinical myocardial dysfunction in pregnant women at higher risk of GDM.

Haplotypes can influence disease development by affecting pathways of cell survival, the immune response, and the regulation of cell death (42). Located on chromosome 6, the HLA region comprise an abundant array of immune-related genes and is, to some extent, also influenced by haplotypes (43). We performed LD analysis on seven SNPs within the *HLA-DQA1/HLA-DQB1* genes, revealing significant LD among these loci. Haplotype analysis indicated that the ACGAATA and ACGGGTA haplotypes may be associated with a reduced risk of GDM, and the ACGAGTA and ACGGATA haplotypes could increase susceptibility to GDM. Compare the database with previous studies, no correlation was found between these haplotypes and other immune or metabolic diseases. Only cyclin-dependent kinase 5 regulatory subunit related protein 1-like 1 and vascular endothelial growth factor gene haplotypes were found to be associated with the risk of GDM (44, 45). These findings may serve as potential biological markers for predicting GDM risk and enhance our understanding of the genetic basis of this disease.

This study explored the association between *HLA-DQA1/DQB1* gene polymorphisms and the risk of GDM, utilizing a relatively large sample size and multiple analytical methods to assess potential interactions. However, there are several limitations in this study. First, as a hospital-based case-control study, there is inherent bias in participant selection and research data recall. Second, although relatively large-size samples were included, however, the lower minor allele frequency of some tested SNPs might limit the statistical power to detect significant associations in some subgroups. Third, the SNPs chosen for this study do not comprehensively cover all variants, which may lead to the omission of some significant variants. Fourth, due to challenges in postpartum follow-up, we were unable to collect reliable postpartum glucose tolerance data. We could not to determine the remission or persistence of GDM, which limits the interpretation of long-term implications of the observed genetic associations. Therefore, more related potential genetic and environmental variables need to be included.

## 5 Conclusion

In summary, the present study revealed that SNPs in the *HLA-DQA1/HLA-DQB1* genes are significantly associated with the risk of GDM via a single locus or joint effects of gene-gene and gene-environment factors. However, larger sample size studies with different populations and related functional experiments are warranted to confirm these positive findings.

## Data Availability

The original contributions presented in the study are included in the article/**Supplementary Material**. Further inquiries can be directed to the corresponding authors.
