# Supplementary material for: Genetic variants in HLA-DQA1/DQB1 genes modulate the risk of gestational diabetes mellitus in a southern Chinese population
Source: Front Endocrinol (Lausanne). 2025 Jul 23;16:1511561. doi: 10.3389/fendo.2025.1511561 (PMC12325038; doi:10.3389/fendo.2025.1511561)
Supplement: Supplementary file 1 [file DataSheet1.docx]

| Loci | Primer Sequence 2nd-PCRP | Primer Sequence 1st-PCRP |
| --- | --- | --- |
| rs1391371 | ACGTTGGATGGTGCTGAGAATCTCAGAAGG | ACGTTGGATGCCGTAAGTTAATGGAGCTTC |
| rs9272425 | ACGTTGGATGGGTTTGGTTTGGGTGTCTTC | ACGTTGGATGTCAGCAGTTGGTGTTCTGAG |
| rs9272426 | ACGTTGGATGTCAGCAGTTGGTGTTCTGAG | ACGTTGGATGGGTTTGGTTTGGGTGTCTTC |
| rs9272460 | ACGTTGGATGTTCAGTGACTACGGCCTGGA | ACGTTGGATGTCCACTGATGGAAACCTCAC |
| rs9273368 | ACGTTGGATGACAAAGGGAGCTCAGACAAG | ACGTTGGATGACAAATGGCAGAGCTCCCTC |
| rs9273505 | ACGTTGGATGAAGTGGGCATCATCCTAGTG | ACGTTGGATGGCCTTAACTTTGGTGGCATC |
| rs9274666 | ACGTTGGATGTGGCAGTGACTTTTTAGCCC | ACGTTGGATGTGTCTCACCTGAATTGCCTG |

Supplementary Table 1. PCR amplified the primer sequence

Supplementary
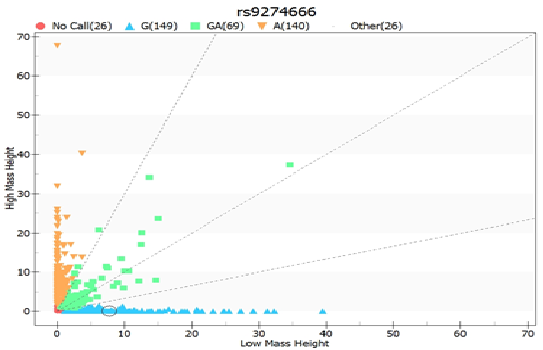

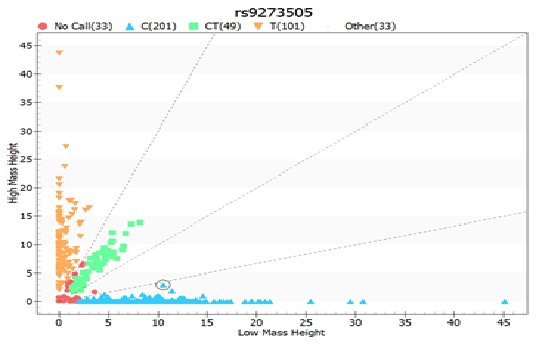

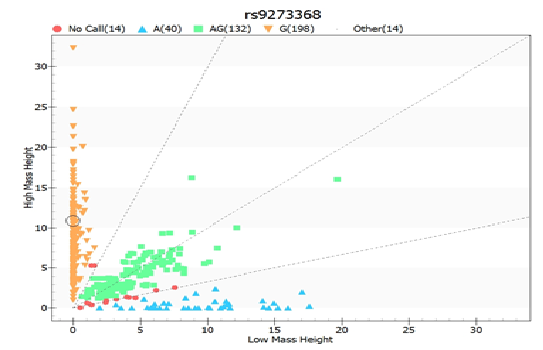

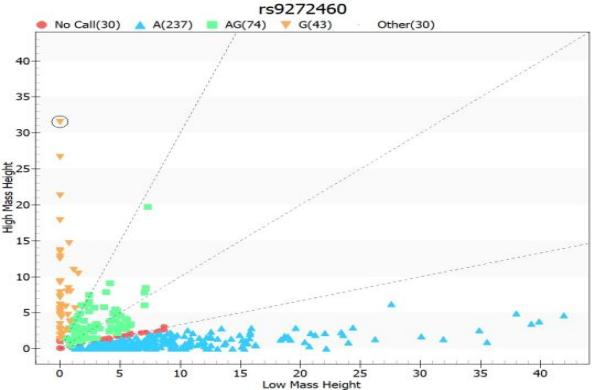

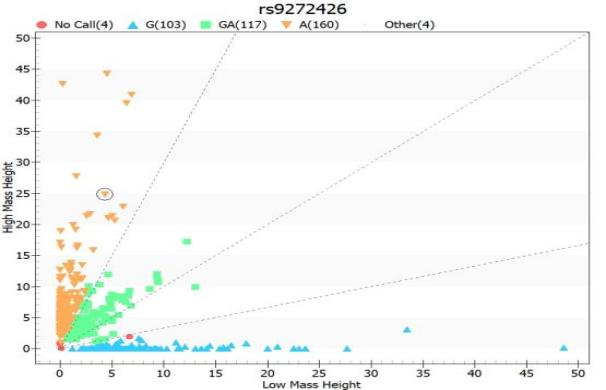

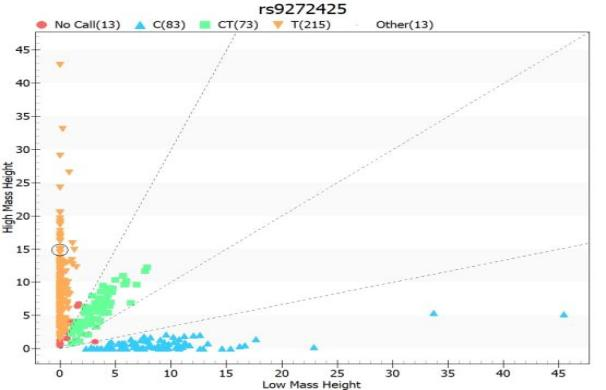

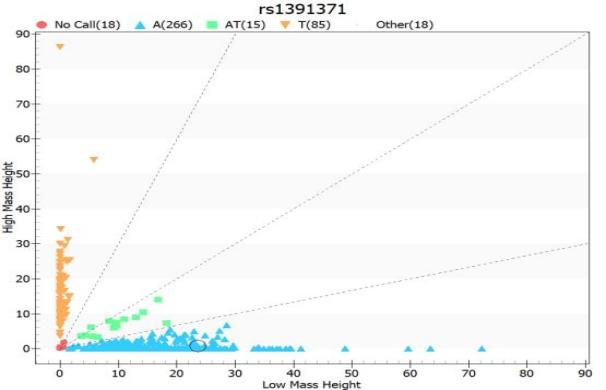
Fig 1. *HLA- DQA1/DQB1* gene candidate genetic variants selection and genotyping
